# Second Hepatectomy Improves Survival in Patients With Microvascular Invasive Hepatocellular Carcinoma Meeting the Milan Criteria

**DOI:** 10.1097/MD.0000000000002070

**Published:** 2015-12-07

**Authors:** Yi-Fu Hou, Bo Li, Yong-Gang Wei, Jia-Yin Yang, Tian-Fu Wen, Ming-Qing Xu, L.V.-Nan Yan, Ke-Fei Chen

**Affiliations:** From the Department of Hepatic Surgery, West China Hospital, Sichuan University, Chengdu, Sichuan Province, China.

## Abstract

Microvascular invasion (MVI) is a strong risk factor for patients with hepatocellular carcinoma (HCC) meeting the Milan criteria and who have received curative hepatectomy. The relevance of a second hepatectomy in patients with MVI-positive recurrent HCC remains controversial.

We had 329 cases of HCC hepatectomy meeting the Milan criteria and compared data on patient demographics, liver function, and tumor pathology between MVI-positive and MVI-negative group. We analyzed potential risk factors of overall survival (OS) and disease-free survival (DFS). Furthermore, newly developed pathological features following the second hepatectomy were also analyzed.

The median OS and DFS were significantly superior in the MVI-negative group than in the MVI-positive group, 61 (10–81) versus 49 (11–82) months (*P* < 0.01) and 41 (7–75) versus 13 (3–69) months (*P* < 0.01), respectively. The presence of MVI and a total tumor diameter >3 cm were independent risk factors associated with both OS and DFS. Overall survival was significantly improved by a second hepatectomy in the MVI-positive group compared with the original MVI-positive group, 60 (26–82) versus 49 (11–82) months, respectively. This was now comparable to the MVI-negative group, 60 (26–82) versus 61 (10–81) months (*P* = 0.72). A second hepatectomy was consistently associated with better survival in the MVI-negative group as compared to the MVI-positive group.

A second hepatectomy improves survival in patients with MVI HCC meeting the Milan criteria. The biology of MVI may change following a second hepatectomy. The absence of MVI is a good prognostic sign for patients undergoing second hepatectomy.

## INTRODUCTION

Hepatocellular carcinoma (HCC) is the most common primary malignant focal liver lesion in the world.^1,2^ It is the sixth most common cancer and the third most common cause of cancer-related death.^3^ In China, chronic infection with hepatitis B is the strongest risk factor for HCC. There are 93 million hepatitis B carriers in China and their risk of developing HCC is ∼200 times higher than that of noncarriers.^4–7^ According to most clinical guidelines, liver resection and liver transplantation represent curative treatments for early stage HCC or HCC meeting the Milan criteria, namely, ≤3 nodules, ≤3 cm, or a single HCC ≤5 cm without macrovascular invasion (MaVI).^8–11^ A 5-year overall survival (OS) >70% and a tumor recurrence in <10% of all cases who receive liver transplantation have been reported.^9^

However, because of the extremely limited number of organ donors in China, transplantation in patients with HCC meeting the Milan criteria is usually not an option. Therefore, hepatectomy remains the only treatment option.^12–14^ Unfortunately, hepatectomy is associated with a relatively high risk of recurrence despite multiple advances in surgical techniques. It has been reported that HCC recurs in 70% to 80% of all cases over the 5 years following curative hepatectomy.^15–17^ Tumor biological characteristics such as pathologically verified MVI has been recognized as an important factor influencing survival.^18,19^ Despite the rigorous requirements of the Milan criteria, the existence of MVI cannot be detected before surgery. According to previous studies, the incidence of MVI depends on various tumor characteristics and is closely associated with tumor diameter.^20–22^ The survival impact of MVI in patients meeting the Milan criteria following HCC hepatectomy is unclear.^23^ Moreover, the role of MVI in recurrent HCC is also uncertain because there are few studies on the clinical significance of MVI in patients with recurrent HCC undergoing a second hepatectomy. Whether a second hepatectomy improves survival remains unclear.^24,25^ The clinical significance of MVI changing after a second hepatectomy warrants clarification.

The present retrospective study was performed to analyze the long-term survival in patients meeting the Milan criteria after hepatectomy and how posthepatectomy MVI status affects survival.

## MATERIALS AND METHODS

### Patients

From January 2007 to December 2012, a total of 970 HCC patients received hepatectomy in West China Hospital of Sichuan University. Patients were included based on the following criteria: (1) HCC meeting the Milan criteria, that is, ≤3 nodules, ≤3 cm, or a single HCC of ≥5 cm without vascular invasion; (2) male or female patient ≥18 years of age; (3) histologically confirmed HCC; (4) liver function status corresponding to Child-Pugh class A or B; (5) total bilirubin ≤1 mg/dL with an Eastern Cooperative Oncology Group performance status of 0 or 1;^26^ and (6) absence of portal hypertension, hypersplenism, and splenomegaly. The exclusion criteria were as follows: (1) margin HCC-positive of the initial resection; (2) a previous history of other malignancy; (3) pathological confirmation of mixed-type HCC with intrahepatic cholangiocellular carcinoma. We included OS, disease-free survival (DFS) time, postrecurrence survival, and postoperative complications (PC) following hepatectomy to be the end points of our study. Our institutional review board approved the study.

### Hepatectomy

Preoperative assessment was essential for patients who were about to receive partial hepatectomy and included blood routine tests, liver function tests, serum alpha-fetal protein (AFP) tests, indocyanine green (ICG) clearance tests, and 3-phase-enhanced computed tomography (CT), or magnetic resonance imaging (MRI) scans. A 15-min ICG retention rate ≤15% for major hepatectomy (≥3 segmentectomies), ≤20% for minor hepatectomy (≤ 2 segmentectomies), and a remnant liver volume ≥50% of the total liver volume were the baseline requirements for hepatectomy. All patients were subject to open surgery. We used Cavitron Ultrasonic Surgical Aspirator or clamp crushing as the 2 principal methods for parenchymal dissection. Continuous/intermittent selective hepatic vascular occlusion was applied to control surgical blood loss. We used intraoperative ultrasonography to guide our resection margin and any additional nodules missed by preoperative imaging studying were also removed. A margin of nontumoral hepatic tissue ≥5 mm was required and ≥1 cm was preferred. We considered anatomic resection preferential to nonanatomic resection.

### Pathological Examination

The histological grade was based on the criteria of the Edmondson–Steiner classification. Therefore, we defined grade I and grade II as well-differentiated and grade III and grade IV as poor-differentiated tumors.^27^ Microvascular invasion was defined as tumor embolus detected by microscopy in the hepatic veins, portal system, and/or lymphatic ducts. Satellite lesions were defined as small lesions within 2 cm of the HCC mass.^19^ Our pathological diagnostic algorithm followed the Chinese guidelines for standardized pathological diagnosis of HCC.^28^ All tumor and nontumoral tissues were examined microscopically.

### Hospitalization and Follow-up

Data on total hospital stay were available through the hospital digital health care system. To compare postoperative complications, we used the Clavien classification to classify the severity.^29^ After initial hepatectomy, the patients were followed up at our outpatient clinic at intervals of 1 to 3 months until recurrence was confirmed. With the help of the local police security system, we could track every patient lost to follow-up. Blood routine tests, liver function tests, serum AFP tests, and abdominal ultrasonography were carried out at each follow-up. When suspicious nodules were found or persistent elevated AFP levels were observed, enhanced CT or MRI would be applied to the patient for confirmation. The diagnosis of recurrent HCC depended on a 3-phase-enhanced imaging study showing typical features of HCC (contrast uptake in the arterial phase followed by rapid washout in the venous phase).^30^

### Treatment of Recurrent HCC

When HCC recurrence was confirmed, repeated hepatectomy was still the first choice if the patient had sufficient hepatic reserve. Radiofrequency ablation (RFA) was used as a substitutional option for resection when the recurrent tumor diameter was within 2 cm. Transarterial chemoembolization (TACE) was the palliative therapy therapeutic option for intrahepatic HCC management.^25^ Chemotherapy and radiation were applied to some patients with extrahepatic HCC lesions. For patients with uncontrollable HCC or bad general condition, best supportive care was carried out. When recurrence occurred after the second hepatectomy, the treatment choice followed the description mentioned above.

### Statistical Analysis

Baseline comparisons between the 2 groups were analyzed with Student's *t* test for continuous variables, the Mann–Whitney *U* test for nonparametric variables, and the chi-square test for categorical variables. Fisher's exact test was applied to compare PC. Cumulative OS rates between the 2 groups were analyzed using the Kaplan–Meier survival curves and tested using the log-rank test. Univariate and multivariate Cox proportional hazard models were used to identify prognostic risk factors. Probability (*P*) values < 0.05 were considered statistically significant. All analyses were performed with SPSS version 21.0 (IBM SPSS Inc, Chicago, IL).

## RESULTS

### Patient Demographics

Among 960 cases of HCC hepatectomy, 329 patients who met the inclusion and exclusion criteria were retrospectively enrolled. There were 102 (31.0%) patients in the MVI-positive group (test group) and 227 (69.0%) patients in the MVI-negative group (control group). The general patient characteristics such as age, male: female ratio, blood test results, and Child–Pugh status were comparable (Table 1). In the test group, patients presented with similar tumor status as compared with the control group (size: 3.5 [2–6] cm vs 4 [1–6] cm and number: 1 [1–3] vs 1 [1–3] [*P* = 0.99 and *P* = 0.73, respectively]). Serum AFP levels were significantly higher in the test group as compared with the control group (160.1 [1.7–1210.0] ng/mL vs 18.7 [1.8–1210.0] ng/mL [*P* < 0.01]). Other parameters of pathological character such as satellite lesions were comparable (11.8% [12/102] vs 11.5% [26/227] [*P* = 0.94]), and the ratio of poorly differentiated cells was significantly higher in the test group than that in the control group (32.4% [33/102] vs16.3% [37/227], *P* < 0.01).

**TABLE 1:**
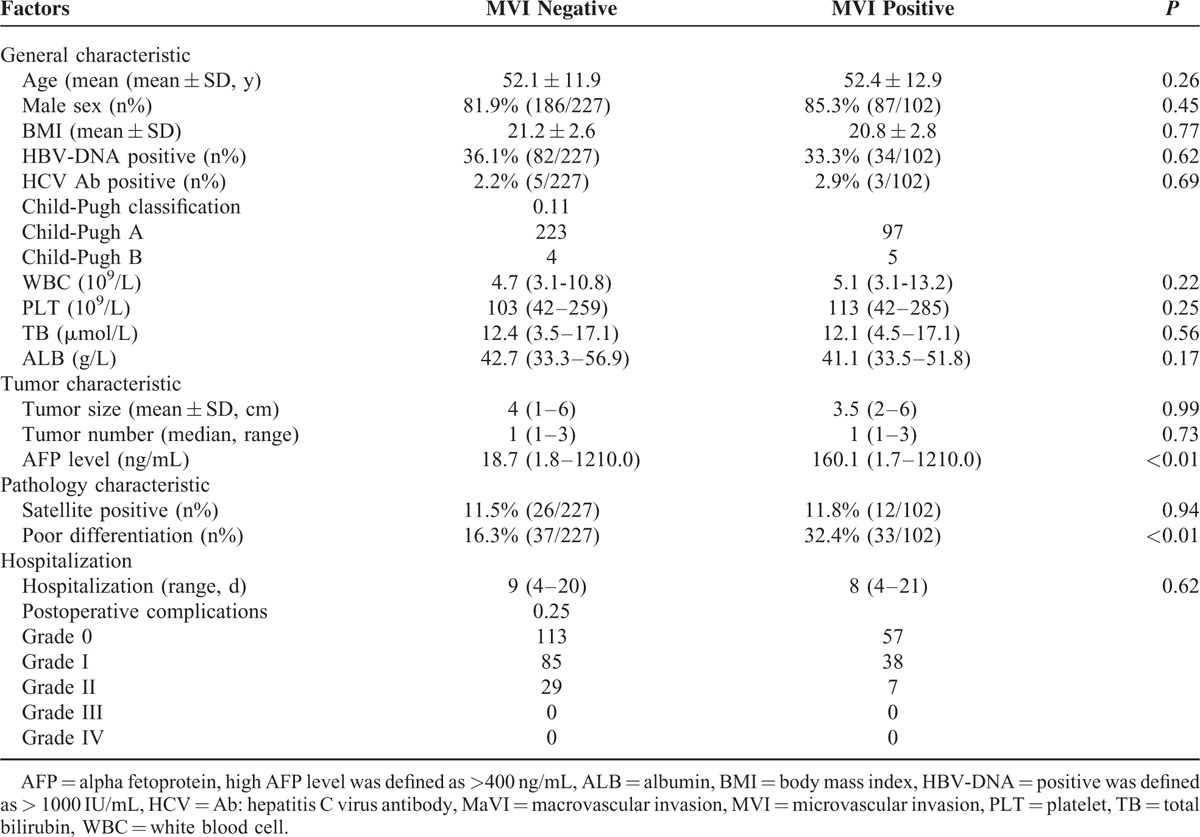
Patients’ Baseline Comparison the MVI-Positive Group and the MVI-Negative Group

### Surgical Outcomes and Postoperative Hospitalization

Postoperative complications were comparable between the 2 groups. There were 57 (55.6%) cases of grade 0 events, 38 (37.5%) cases of grade I events, and 7 (6.9%) cases of grade II events in the test group and the corresponding results for the control group were 113 cases (48.9%), 85 cases (37.4%), and 29 cases (12.7%), respectively (*P* = 0.25). No cases of severe complication or hospital-associated mortality were recorded in any of the groups. Median lengths of hospitalization were comparable between the test and control groups, 8 (4–21) days versus 9 (4–20) days (*P* = 0.62; Table 1).

### Survival

**FIGURE 1:**
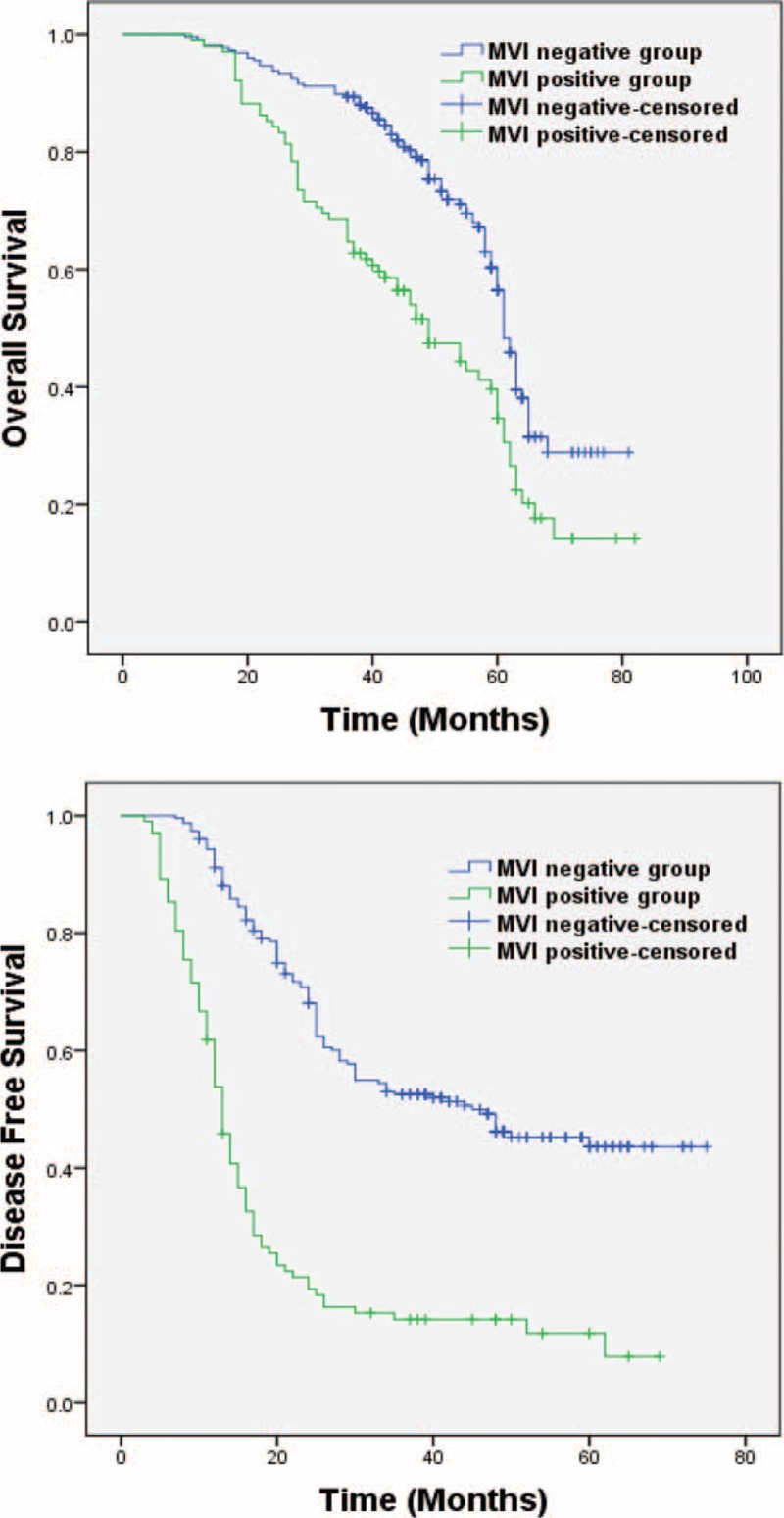
OS and DFS between the test group and the control group, *P* < 0.01, *P* < 0.01, respectively. DFS = disease-free survival; OS = overall survival.

The median follow-up time was 60 (10–82) months. The median OS time and DFS in the test and control groups were 49 (11–82) months versus 61 (10–81) months and 13 (3–69) months versus 41 (7–75) (*P* < 0.001 and *P* < 0.001), respectively (Figure 1). The 1-, 3-, and 5-year DFS rates in the test group were 53.8%, 14.2%, and 11.8%, respectively. The corresponding data in the control group were 91.2%, 52.5%, and 43.6%, respectively. The 1-, 3-, and 5-year OS rates in the test group were 98.0%, 64.7%, and 34.6%, respectively. The corresponding data in the control group were 99.1%, 89.4%, and 56.4%, respectively. At the end of our study, a total of 68 deaths had been recorded in the test group. Two died of liver cirrhosis, 1 of rejection reaction after salvage transplantation, 1 of an accident, and the remaining 64 because of cancer. In the control group, there were 93 deaths; 24 died of liver cirrhosis, 1 died of chronic renal failure, and the remaining 68 deaths were attributed to cancer. By the end of the follow-up, 14 cases were recurrence-free in the test group, whereas 88 cases experienced HCC recurrence. Among the 88 cases, 45 received a second hepatectomy, 33 received TACE, 9 received RFA, and 1 received salvage transplantation. In the control group, 112 cases were recurrence-free, and 115 cases experienced HCC recurrence. Among the latter, 85 received a second hepatectomy, 18 received RFA, and 12 received TACE.

**FIGURE 2:**
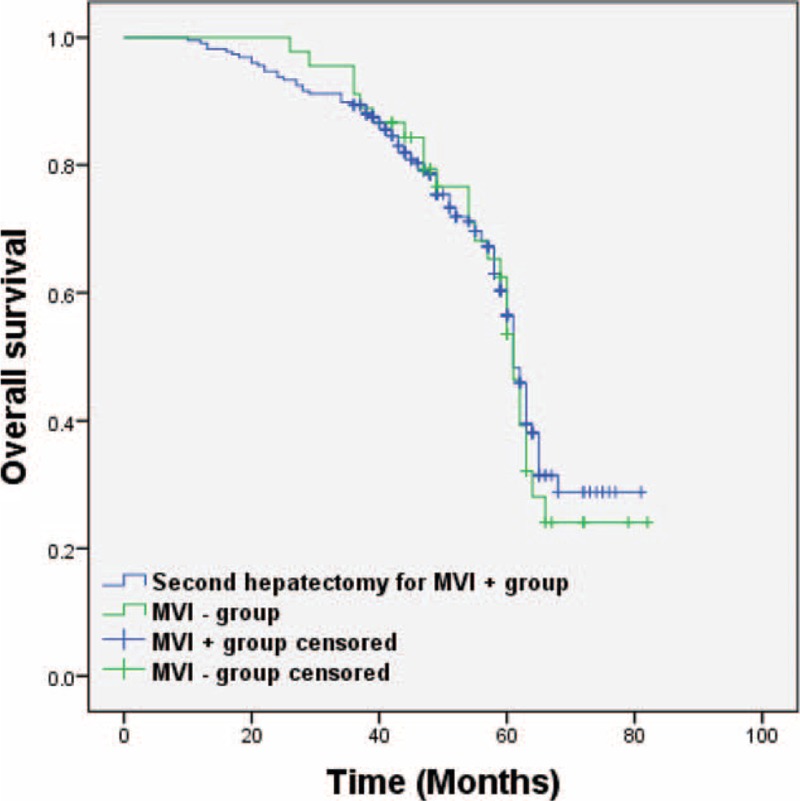
OS between second hepatectomy in the MVI-positive group and the MVI-negative group, *P* = 0.72. OS = overall survival.

**FIGURE 3:**
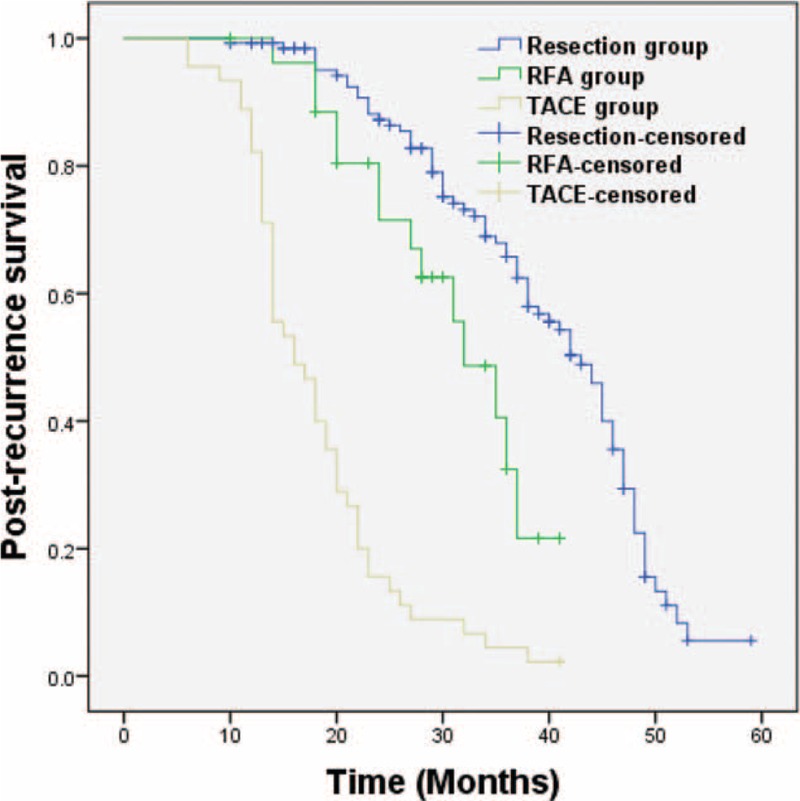
Second hepatectomy improved survival after recurrence as compared to RFA and TACE, *P* = 0.01, *P* < 0.01, respectively. RFA = radiofrequency ablation; TACE = transarterial chemoembolization.

In a subgroup analysis, we compared the OS in patients who received a second hepatectomy because of HCC intrahepatic recurrence in the MVI-negative and the MVI-positive groups. The results showed a comparable outcome between the MVI-positive group and the MVI-negative group: 61 (26–82) months versus 61 (10–81) months (*P* = 0.72; Figure 2). Furthermore, we compared the different interventions used for treating recurrent HCC and found that postrecurrence OS was significantly improved after the 2nd hepatectomy as compared with RFA and TACE, 43 (10–59) months versus 32 (10–41) months and 43 (10–59) months versus 16 (6–41) months (*P* = 0.01 and *P* < 0.01), respectively (Figure 3).

**FIGURE 4:**
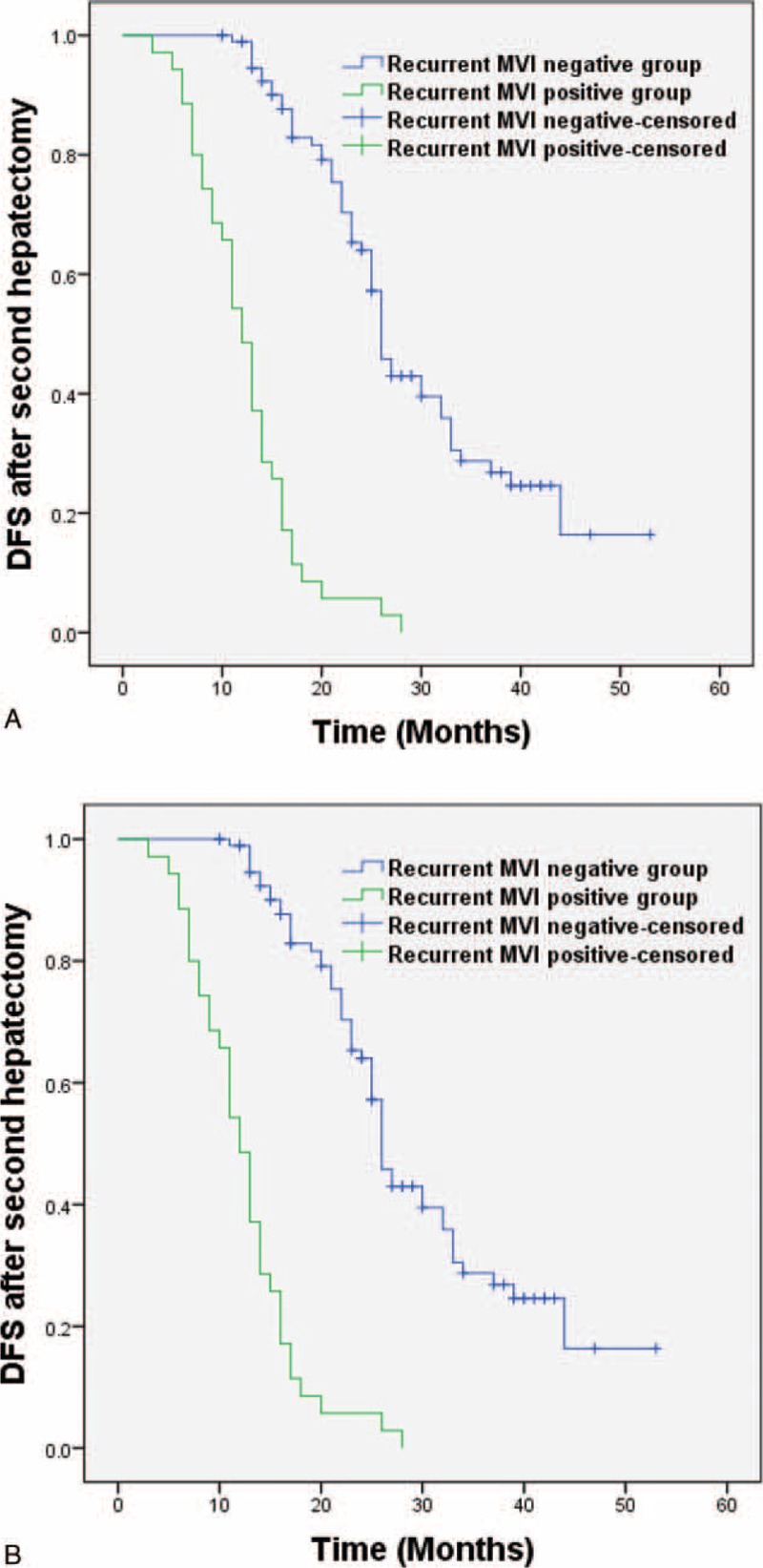
Disease-free survival after second hepatectomy between the new MVI-positive group and the new MVI-negative group, *P* < 0.01. MVI = microvascular invasion.

For patients who received a second hepatectomy, we noticed that some had different MVI results as compared with the first hepatectomy. In total there were 11 (8.5%) MVI positive-negative shifting cases and 24 (18.5%) MVI negative–positive shifting cases. Moreover, a comparison of DFS following second hepatectomy for patients in the MVI-positive and MVI-negative groups with regard to the results of second pathological examination showed that the new MVI-positive group had a significantly worse outcome than the new MVI-negative group, 13 (3–28) months versus 26 (10–53) months (*P* < 0.01; Figure 4).

### Prognostic Factors

Table 2 lists the results of univariate and multivariate analyses of prognostic factors related to OS and DFS for all study individuals. The factors significantly associated with OS identified by univariate analysis included total tumor diameter >3 cm (hazard ratio [HR] = 1.50, *P* = 0.01) and MVI positivity (HR = 2.02, *P* < 0.01). For multivariate analysis, we selected factors identified using univariate analysis with *P* values < 0.05 together with certain significant clinical variables. The significant risk factors related to worse OS identified using multivariate analysis were as follows: MVI positivity (HR = 2.04; 95% confidence interval [CI], 1.49–2.80; *P* < 0.01), and total tumor diameter >3 cm (HR = 1.49; 95% CI, 1.09–2.06; *P* = 0.01) (Table 2).

**TABLE 2:**
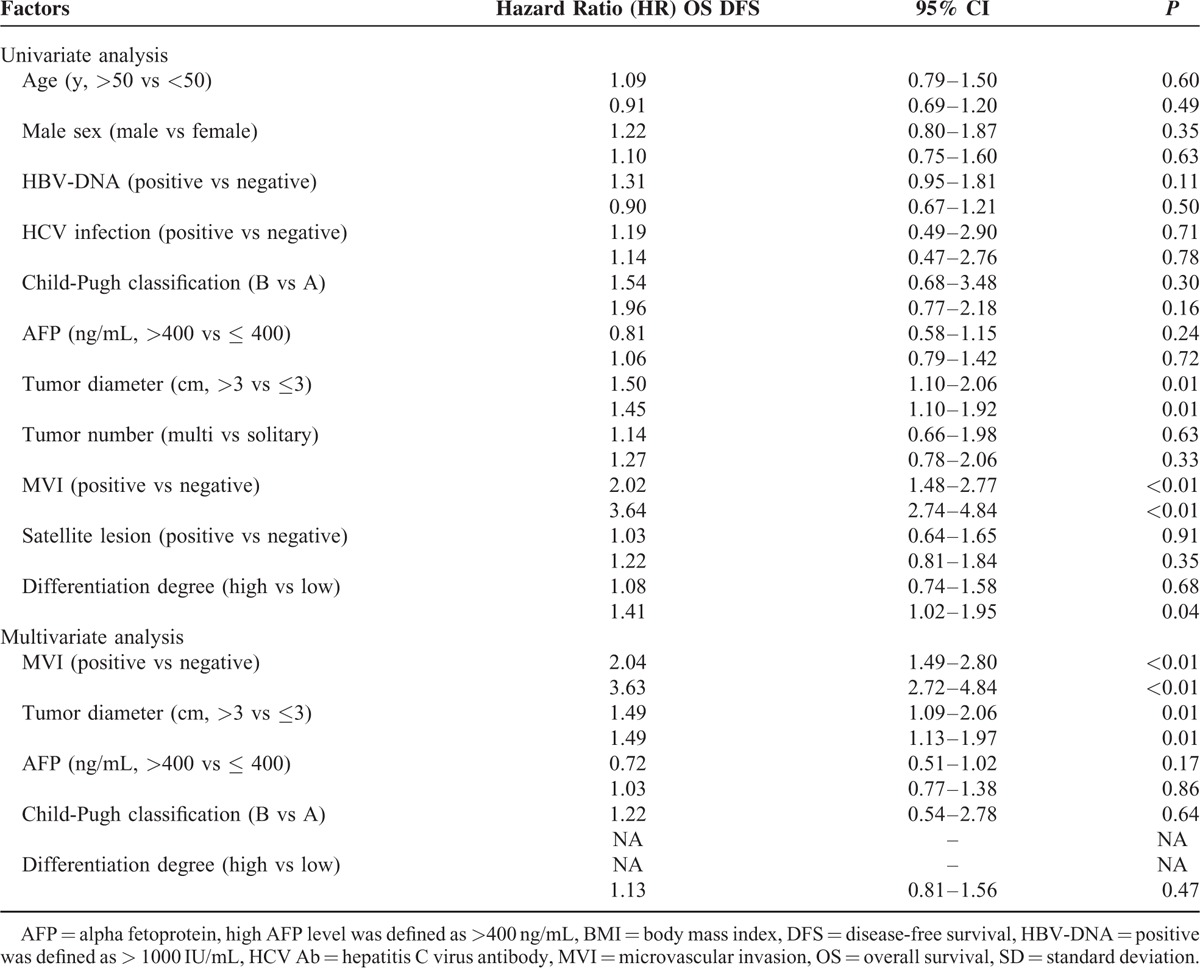
Univariate and Multivariate Analysis for Prognostic Factors of OS and DFS

Prognostic factors of DFS as revealed by univariate analysis included a total tumor diameter >3 cm (HR = 1.45, *P* = 0.01), MVI positivity (HR = 3.64, *P* < 0.01), and low differentiation degree (HR = 1.41, *P* = 0.04). Factors identified using univariate analysis with *P* values < 0.05 together with certain significant clinical variables were also selected for multivariate analysis. This revealed that significant risk factors related to DFS were MVI (HR = 3.63; 95% CI, 2.72–4.84; *P* < 0.01) and total tumor diameter (HR = 1.49; 95% CI, 1.13–1.97; *P* = 0.01) (Table 2).

## DISCUSSION

Hepatocellular carcinoma meeting the Milan criteria is generally considered to be early HCC with a relatively high potential for cure. According to the criteria, the 5-year mortality is expected to be < 30% for patients undergoing liver transplantation.^8,9,31^ However, because of the large number of hepatitis B carriers and the extremely limited number of organ donors in China, most patients meeting the Milan criteria still receive partial hepatectomy. In this study, the estimated median survival for all the patients reached 60 months, reflecting a generally satisfying result of disease control. This result is also consistent with the data of stage A HCC of Barcelona clinic liver cancer staging system.^32^ However, despite the early stage of HCC, the estimated median progression-free survival for all patients after the curative resection was 25 (3–75) months, which was worse than the outcome of liver transplantation.^9^ Tumor behavior in early stage HCC warrants further understanding. This article focuses on the influence of an important tumor biological behavior, microvascular invasion, and on patient survival.

Appearing when a tumor starts metastasizing, MVI represents an aggressive behavior of HCC.^33,34^ Microvascular invasion is closely related to the morphological features of the tumor such as size, number, and so on. According to a previous study, the likelihood of MVI development increases as the tumor becomes larger.^20^ In our study, among 327 cases of HCC meeting the Milan criteria, 102 cases were MVI-positive. The MVI rate was 31.2%, which is surprisingly high considering the fact that the tumor is generally considered less aggressive at this early stage. Baseline comparisons showed that the MVI-positive group had almost the same tumor status and pathological results as the MVI-negative group, except for higher serum AFP levels and lower differentiated tumors. Theoretically, a lower degree of differentiation reflects higher immature tumor cell immaturity along with a higher likelihood of development of peri-tumor or distant invasion. Moreover, the serum AFP level is associated with certain subtypes of the HCC, and based on early basic studies and recent clinic findings, considered to be a potential risk factor of survival.^35^ Therefore, it makes sense those MVI-positive patients present with lower differentiated tumors and higher AFP levels. In the survival analysis, the test group unsurprisingly showed both worse OS and DFS than the control group. In more detail, ∼50% of the patients in the test group experienced HCC recurrence within the first year following hepatectomy and this proportion had reached ∼80% by the third year. On the other hand, < 10% of the patients in the control group experienced HCC recurrence within the first year following hepatectomy. After 3 years, this share had reached ∼50%. This phenomenon may be explained by earlier studies suggesting that the presence of MVI is related to the time of HCC recurrence, which was reconfirmed by our results.^31,36,37^ According to Llovet's classification, recurrence is defined by (1) true metastasis recurring within 2 years and (2) multicentric tumors that arise *de novo* in a pre-neoplastic liver. Therefore, it is not surprising that MVI is repeatedly identified as a risk factor for early HCC recurrence.^38^ Moreover, the results of our multivariate analyses showed that both total tumor diameter >3 cm and MVI are the independent risk factors of OS and DFS. These results suggest that size matters in early stage HCC and special attention should be paid to both MVI and tumor burden in the management of early stage HCC.

The management of recurrent HCC is still controversial.^24,25^ In China, we value the importance of repeat hepatectomy, especially in patients with primary early stage HCC. In this study, second hepatectomy was chosen as the first choice for recurrent HCC. Our results showed that patients in the test group who received a second hepatectomy had a comparable outcome to that observed in patients in the control group. Hence, second hepatectomy improves survival in HCC MVI-positive patients meeting the Milan criteria.^38^ Interestingly, with regard to the pattern of MVI changes following second hepatectomy, we observed 24 MVI-negative shifting cases and 11 MVI-positive shifting cases. This phenomenon of MVI shifting had a significant influence on patient survival (Figure 4). More specifically, 33/35 (94.3%) MVI-positive cases following the second hepatectomy experienced recurrence once again within 2 years (early recurrence). However, only 32/95 (33.7%) MVI-negative cases following the second hepatectomy experienced early recurrence. A possible explanation is that the 21 MVI-negative shifting cases and 11 MVI-positive shifting cases may reflect tumor phenotype differences and not tumor intrahepatic spread. We have previously shown that some early stage MVI-positive cases of HCC could show alternative results on MVI following a second haptectomy.^39^ In this article, we focused on early stage HCC and obtained consistent results. For diagnostic accuracy, we value the importance of the pathological results of HCC, especially early stage HCC. Complete examination of all tumor and nontumor tissue is mandatory for small HCC. Although MVI is associated with poor patient survival, the presence of MVI-negative shifting may suggest the complete clearance of the tumor. Hence, MVI could be a sensitive marker for patient outcome—whenever MVI disappears, a better prognosis can be expected.

In the management of HCC recurrence, we find that repeat hepatectomy provides better survival than RFA and TACE. Thanks to advances in the surgical technique and perioperative management over the past few decades, repeat hepatectomy is considered safe for suitable patients.^24,40^ For patients with resectable recurrent HCC, repeat hepatectomy should be considered the first-line choice. However, the results of RFA and TACE in resectable HCC remain unknown because as stated in the Materials and Methods section, only those recurrent patients who were not suitable for a second hepatectomy were otherwise considered for RFA and TACE. Nevertheless, resection has been proven to be a better choice than RFA and TACE for early stage nonrecurrent HCC.^30^ We consider radical resection to be the best option for early stage recurrent HCC.

Our study had some limitations. The retrospective nature and the single-center status of this study limit its validity and further studies are required to confirm our results.

## CONCLUSIONS

Performing a second hepatectomy is a safe and effective approach for treating intrahepatic recurrence of HCC meeting the Milan criteria. It improves survival for patients positive for MVI after the first hepatectomy. The importance of tumor MVI status following second hepatectomy should be acknowledged.
